# Comparison of Ultrasound-Guided Costoclavicular Brachial Plexus Block Versus Supraclavicular Brachial Plexus Block for Forearm and Hand Surgery: A Randomized Controlled Trial

**DOI:** 10.7759/cureus.94648

**Published:** 2025-10-15

**Authors:** Laxman Kumar Senapati, Rajendra Sahoo, Priyadarsini Samanta, Subhadra Priyadarshini

**Affiliations:** 1 Department of Anaesthesiology, Kalinga Institute of Medical Sciences, Bhubaneswar, IND; 2 Department of Anaesthesiology and Pain Medicine, Kalinga Institute of Medical Sciences, Bhubaneswar, IND; 3 Department of Physiology, Kalinga Institute of Medical Sciences, Bhubaneswar, IND; 4 Department of Research & Development, Kalinga Institute of Medical Sciences, Bhubaneswar, IND

**Keywords:** costoclavicular brachial plexus block, forearm and hand surgery, peripheral nerve block, postoperative analgesia, supraclavicular brachial plexus block, ultrasound-guided regional anesthesia

## Abstract

Background

Ultrasound-guided brachial plexus block (BPB) is a cornerstone of anesthesia for upper limb surgeries, with supraclavicular (SC) and costoclavicular (CC) approaches being widely used. The CC approach, a modification of the infraclavicular technique, offers a more clustered neural target and potentially faster block onset with fewer complications.

Objective

This study aims to compare the block characteristics, analgesic efficacy, and safety of ultrasound-guided costoclavicular and supraclavicular brachial plexus blocks in patients undergoing forearm and hand surgery.

Methods

In this prospective, randomized controlled trial, 64 adult patients (American Society of Anesthesiologists (ASA) I-III) scheduled for elective forearm and hand surgery were randomized into two groups: CC-BPB (n = 32) and SC-BPB (n = 32). Both groups received 20 mL of 0.5% ropivacaine under ultrasound guidance. The primary outcome was the onset time of motor block. Secondary outcomes included onset time of sensory block, block performance time, duration of sensory and motor blockade, duration of analgesia, postoperative pain scores, and complications. The Mann-Whitney U test was used for continuous variables, and the chi-square test or Fisher’s exact test for categorical variables. Postoperative VAS scores were analyzed using the Friedman test for repeated measures within each group, followed by Bonferroni-adjusted pairwise comparisons to identify time points with significant differences. The study was registered with the Clinical Trials Registry of India (CTRI/2022/03/041029, dated March 11, 2022).

Results

The CC-BPB group demonstrated a significantly shorter block performance time (1.53 vs. 1.98 minutes, p < 0.001), faster onset of sensory block (9 vs. 10 minutes, p = 0.001), and quicker onset of motor block (12 vs. 13 minutes, p = 0.001) compared to the SC-BPB group. There were no significant differences between groups in the duration of sensory block, motor block, analgesia, or time to first rescue analgesic request. Postoperative pain scores and complication rates were comparable across groups.

Conclusion

Ultrasound-guided costoclavicular brachial plexus block offers faster block onset and shorter performance time than the supraclavicular approach while providing equivalent analgesic duration and safety. CC-BPB represents a reliable alternative to SC-BPB for forearm and hand surgery.

## Introduction

Regional anesthesia (RA) offers enhanced perioperative analgesia, reduced systemic side effects such as nausea, vomiting, and confusion, as well as expedited mobilisation compared to general anesthesia (GA) [1]. Effective peripheral nerve block (PNB) can prevent complications linked to GA, including airway challenges, postoperative respiratory issues, and hemodynamic instability [1]. Ultrasound-guided supraclavicular (SC) and infraclavicular (IC) brachial plexus blocks (BPPBs) have gained popularity for upper-extremity surgery. This is attributed to the enhanced safety provided by real-time ultrasound guidance and quicker onset times [2]. The SC-BPB has withstood the test of time as the primary modality for providing anesthesia and analgesia during upper limb surgery. In SC-BPB, the brachial plexus around the subclavian artery is blocked. As the block was performed above the clavicle, there is a high likelihood of phrenic nerve blockades and consequent hemidiaphragmatic paralysis [3]. SC-BPB is also associated with ulnar nerve sparing and pneumothorax [4].

The costoclavicular approach to BPB (CC-BPB) is a modification of the ultrasound-guided IC-BPB. It was first described by Karmakar et al. and has gained popularity [5]. The CC-BPB facilitates a swift onset of sensory-motor block by targeting the centre of the three neural cords, positioned laterally to the axillary artery (AA) within the CC space. The three cords maintain a reliable anatomical relationship with the AA [6]. CC-BPB differs from SC-BPB in that all three cords of the brachial plexus are clustered in the former, and the low dose of local anesthetic (LA) and single injection provides adequate analgesia and anesthesia [6]. There is a low risk of vessel rupture and pleural puncture in the costoclavicular variant of infraclavicular brachial plexus block, as the nerve cords are first approached before the vessel and the pleura, when compared with other approaches to IC-BPB [3].

There is a paucity of trials comparing the efficacy of ultrasound-guided CC-BPB and SC-BPB in upper extremity surgery. Hence, we undertook this randomized study of ultrasound-guided CC-BPB with conventional SC-BPB to determine the efficiencies, advantages, and disadvantages of the two approaches. We hypothesized that CC-BPB may offer superior block characteristics compared to SC-BPB, based on the anatomical proximity and compact arrangement of the three neural cords lateral to the axillary artery within the costoclavicular space. This configuration allows more homogeneous local anesthetic spread and efficient single-injection blockade, which has been associated with faster onset and reliable anesthesia in prior studies [7,8]. The primary objective was to evaluate the time of onset of sensory and motor blockade between the two approaches. The secondary objectives were to determine the duration of sensory and motor block, the duration of analgesia, the block performance time, and the side effects.

## Materials and methods

Study design, ethical consideration, and trial registration

This was a randomized controlled trial conducted in a tertiary care hospital, Kalinga Institute of Medical Sciences, Bhubaneswar, Odisha, India, between April 2022 and April 2024. The study was approved by the Institutional Ethical Committee of Kalinga Institute of Medical Sciences, Kalinga Institute of Industrial Technology (KIIT), Bhubaneswar (approval number: KIIT/KIMS/IEC/783/2021, dated December 13, 2021) and registered with the Clinical Trials Registry of India (registration number: CTRI/2022/03/041029, dated March 11, 2022). The protocol adhered to the 2013 Declaration of Helsinki and the International Conference on Harmonization’s Good Clinical Practice guidelines. Written informed consent was taken from all participants.

Eligibility criteria 

Inclusion criteria were patients aged 18 to 70 years, American Society of Anesthesiologists (ASA) physical status I to III, undergoing surgeries on the elbow, forearm, wrist, and hand. Exclusion criteria included local site infection, patients with chronic pain and taking opioids, previous infraclavicular fossa surgery, pre-existing neuropathy, clinically significant coagulopathy, pregnancy, and body mass index ˃ 30 kg/m².

Sample size calculation

The sample size was calculated with reference to a previous study by Ramesh et al. (2021) [8], and the mean ± SD of the onset of motor block (in minutes) was 11.72 ± 0.79 for CC-BPB and 12.56 ± 0.92 for SC-BPB, which would be statistically significant. Assuming these reference values, the minimum required sample size at 5% level of significance, 95% power, and 95% confidence interval was at least 29 in each group. The total sample size was thus taken as 64, accounting for a 10% attrition rate.

Randomization, allocation concealment, and blinding

An unbiased researcher with no role in interventions or assessments performed the randomization and concealment process. Patients were randomized into two groups using computer‐generated codes. The researcher, who performed all the evaluations, and the subjects were unaware of the randomization. The allocation sequence was conducted using sequentially numbered, sealed, and opaque envelopes for assignment lists. Each envelope was opened only after the participant consented to join the study, ensuring the allocation was concealed until the point of assignment. Before the block, the interventionist opened the concealed envelope and revealed the patient’s treatment allocation. A nurse prepared the drug, unaware of the study allocation or evaluation process. A different anesthesiologist, blinded to the group allocation and interventions, collected postoperative data.

Intervention

Group 1 (CC-BPB) received an ultrasound-guided costoclavicular brachial plexus block with 20 ml of 0.5% ropivacaine. Group 2 (SC-BPB) received an ultrasound-guided supraclavicular brachial plexus block with 20 ml of 0.5% ropivacaine.

Sonography-Guided Block

An anesthesiologist with expertise in RA and musculoskeletal ultrasound performed all the blocks in the preoperative facility of the operation theatre (OT) complex under ultrasound guidance (SonoSite Edge II; FUJIFILM Sonosite, Bothell, Washington, United States) using a linear high-frequency (6-13 MHz) probe. Standard Indian Society of Anaesthesiologists (ISA) monitoring was performed during block performance, including electrocardiograms, non-invasive blood pressure measurements, and pulse oximetry.

**Figure 1:**
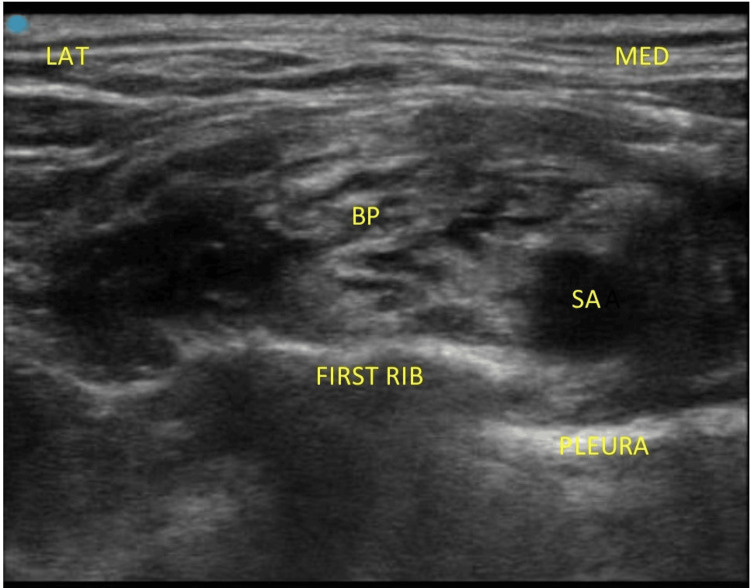
Sono-anatomy of supraclavicular brachial plexus block. MED: medial; LAT: lateral; SA: subclavian artery; BP: brachial plexus elements

**Figure 2:**
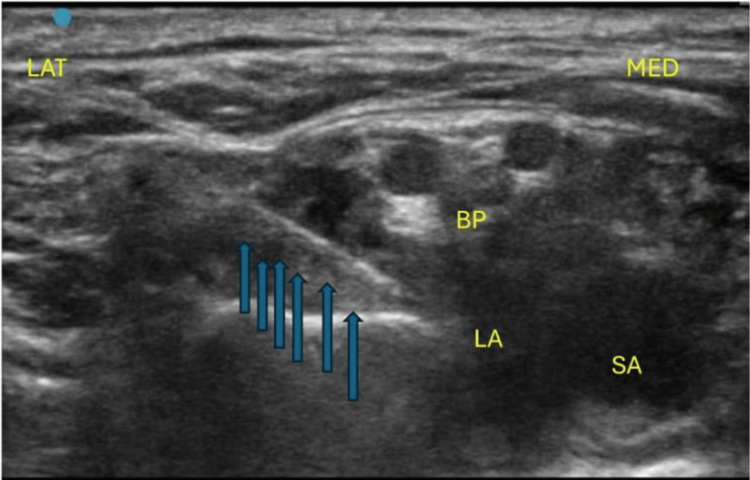
Needle direction (blue arrows) from the lateral to the medial direction in an in-plane manner targeting the corner pocket. MED: medial; LAT: lateral; SA: subclavian artery; BP: brachial plexus elements

**Figure 3:**
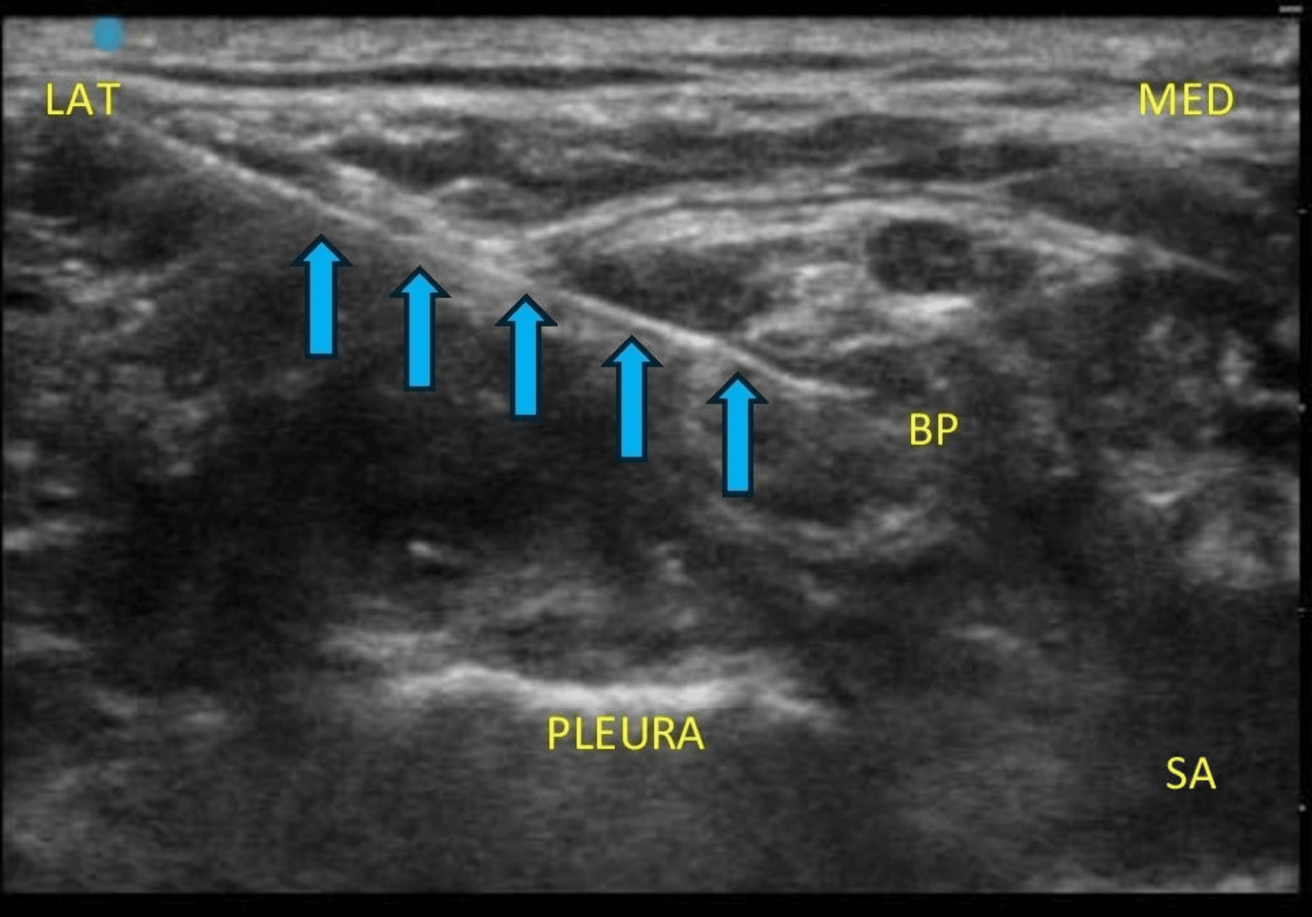
Needle direction (blue arrows) from the lateral to the medial direction in an in-plane manner targeting the midpoint of the brachial plexus. MED: medial; LAT: lateral; SA: subclavian artery; BP: brachial plexus elements

**Figure 4:**
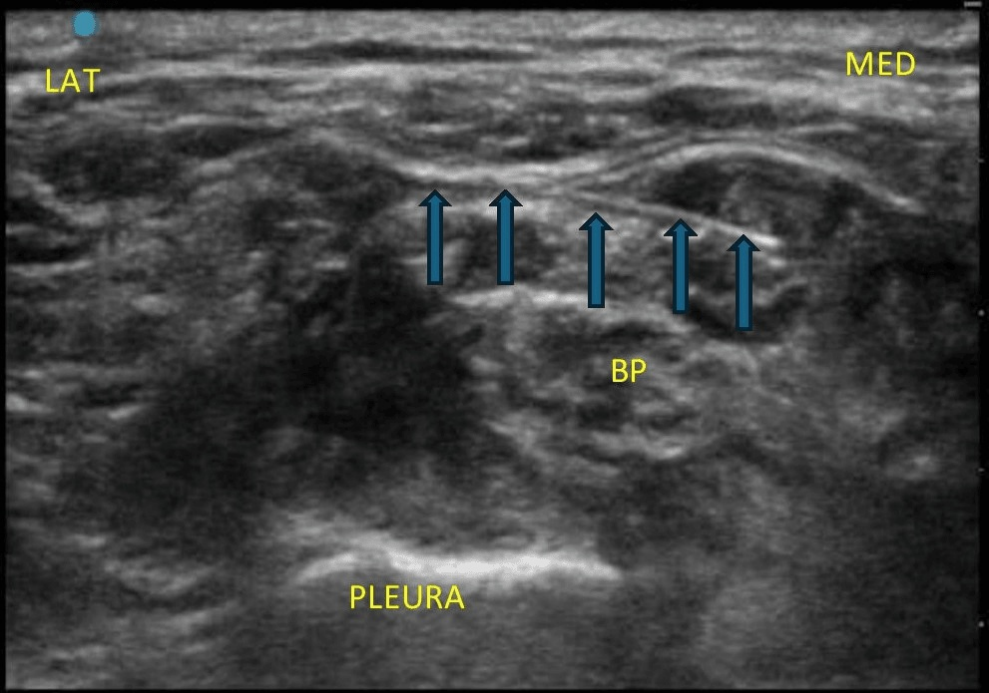
Needle direction (blue arrows) from the lateral to the medial direction in an in-plane manner targeting the superior part of the brachial plexus. MED: medial; LAT: lateral; SA: subclavian artery; BP: brachial plexus elements

Supraclavicular block:* *The transducer was placed in the transverse plane closest to the middle of the clavicle. The brachial plexus appeared as a series of hypoechoic oval structures located superficial to the subclavian artery posteriorly. Using an in-plane approach, the needle was inserted from the lateral to the medial direction. Local anaesthetic was first deposited in the corner pocket, followed by small aliquots at the midpoint and superior aspect of the connective tissue sheath containing the brachial plexus, using two gentle needle redirections without withdrawing the needle. This ensured uniform spread of anaesthetic around the plexus (Figures 1-4) [4].

**Figure 5:**
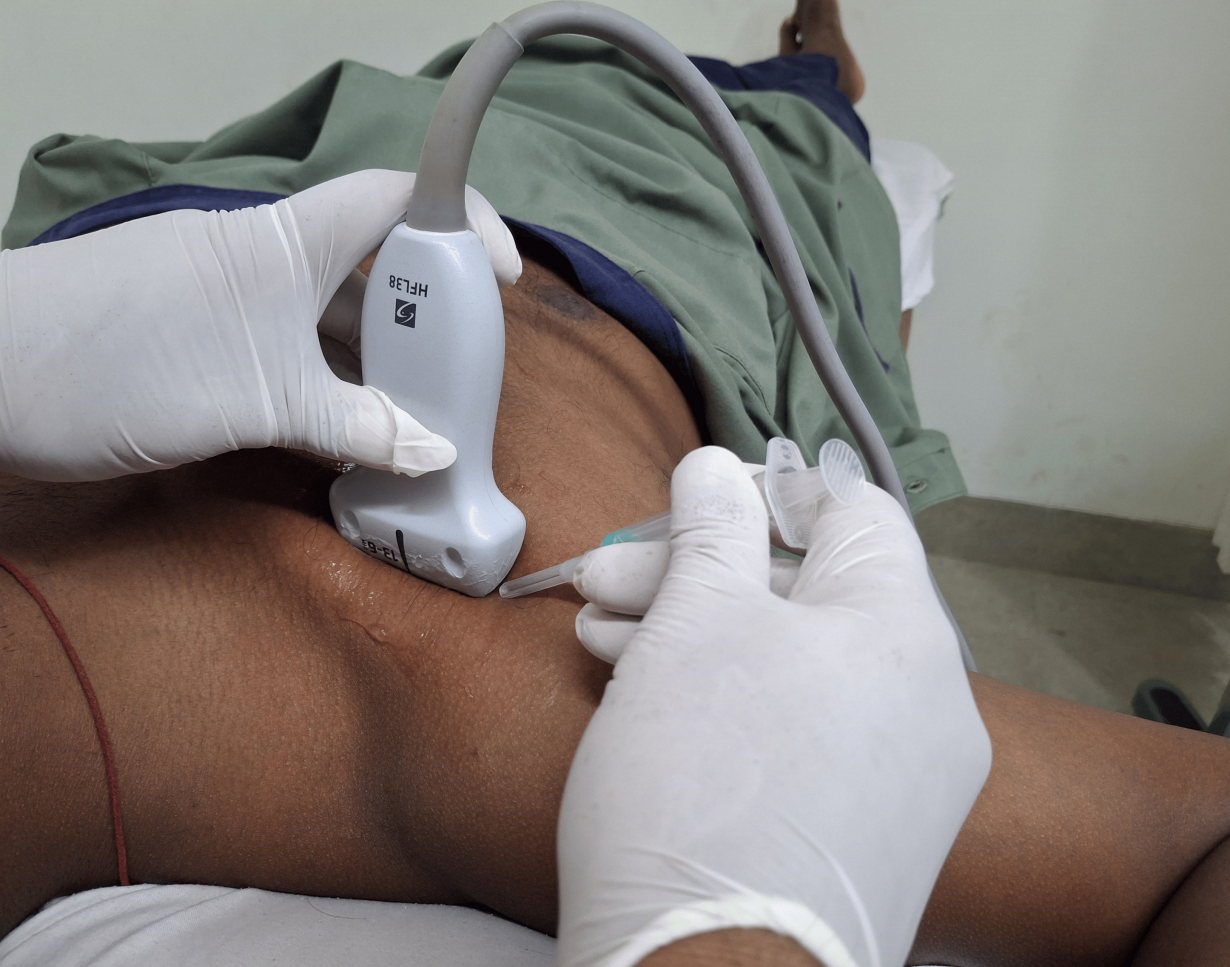
Patient position and orientation of the probe.

**Figure 6:**
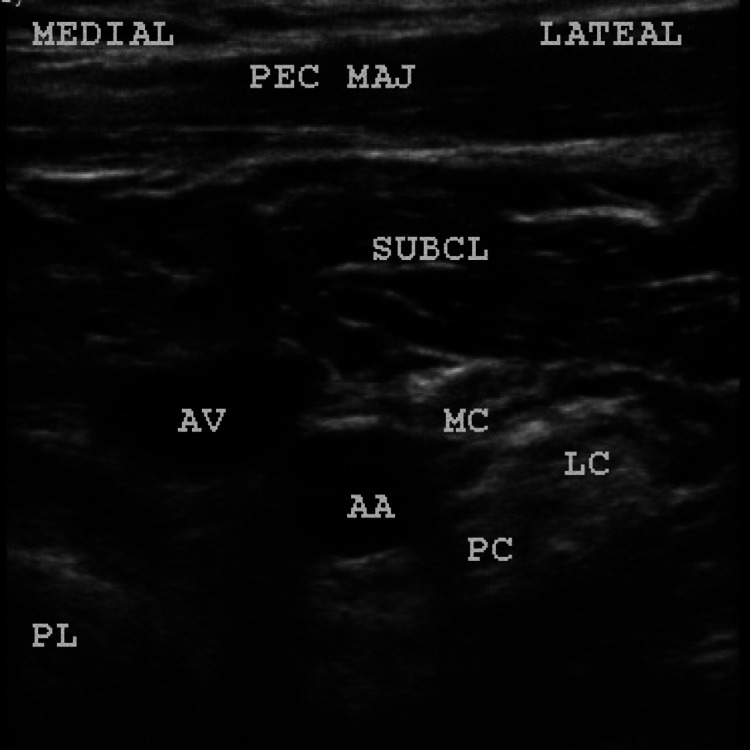
Sono-anatomy of the costoclavicular block. PEC MAJ: pectoralis major; SUBCL: subclavius; AA: axillary artery; AV: axillary vein; Pl: pleura; MC: medial cord; LC: lateral cord; PC: posterior cord

**Figure 7:**
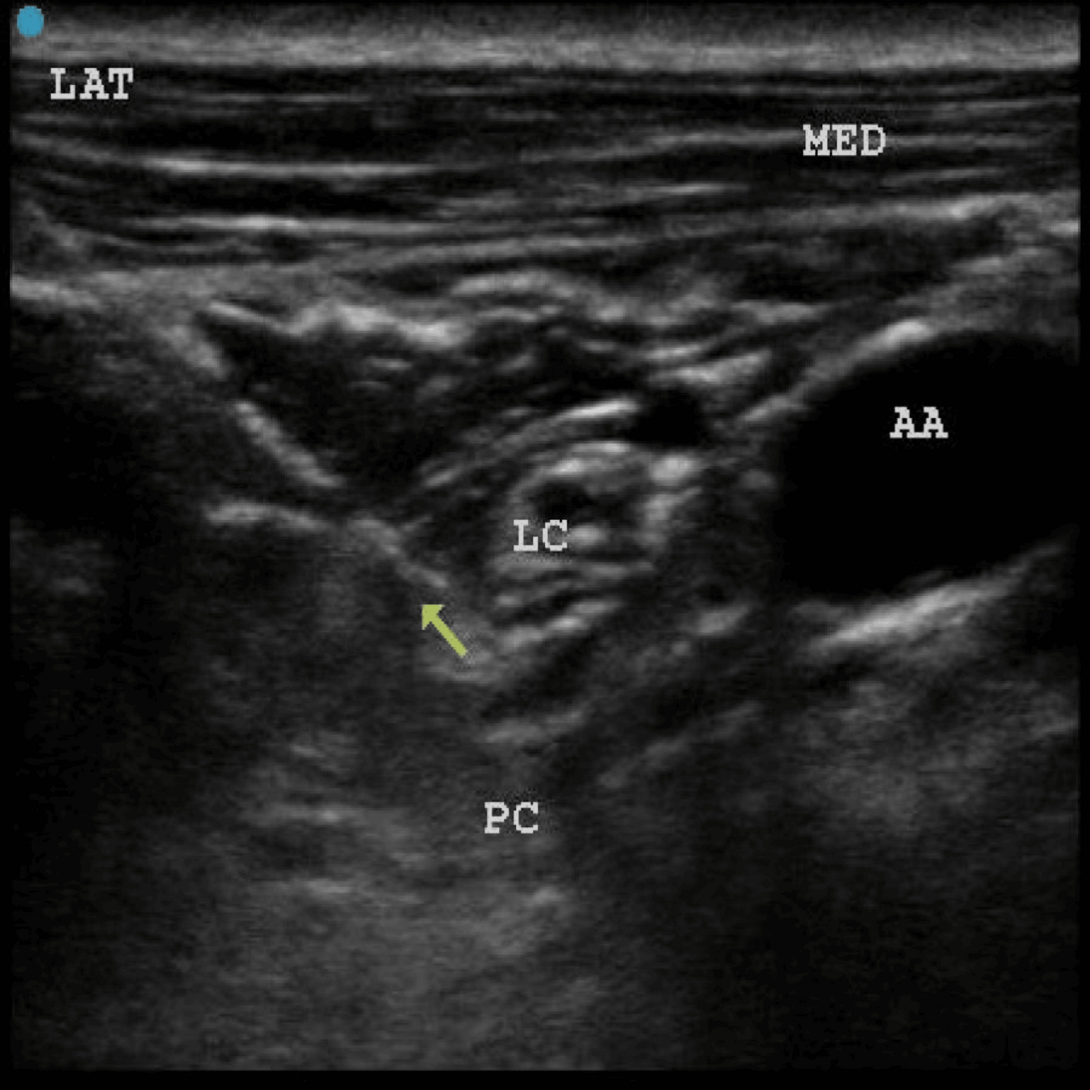
Needle direction from the lateral to the medial direction in an in-plane manner, positioning the needle tip (indicated by yellow arrow) between the three cords. AA: axillary artery; LC: lateral cord; PC: posterior cord; LAT: lateral; MED: medial

**Figure 8:**
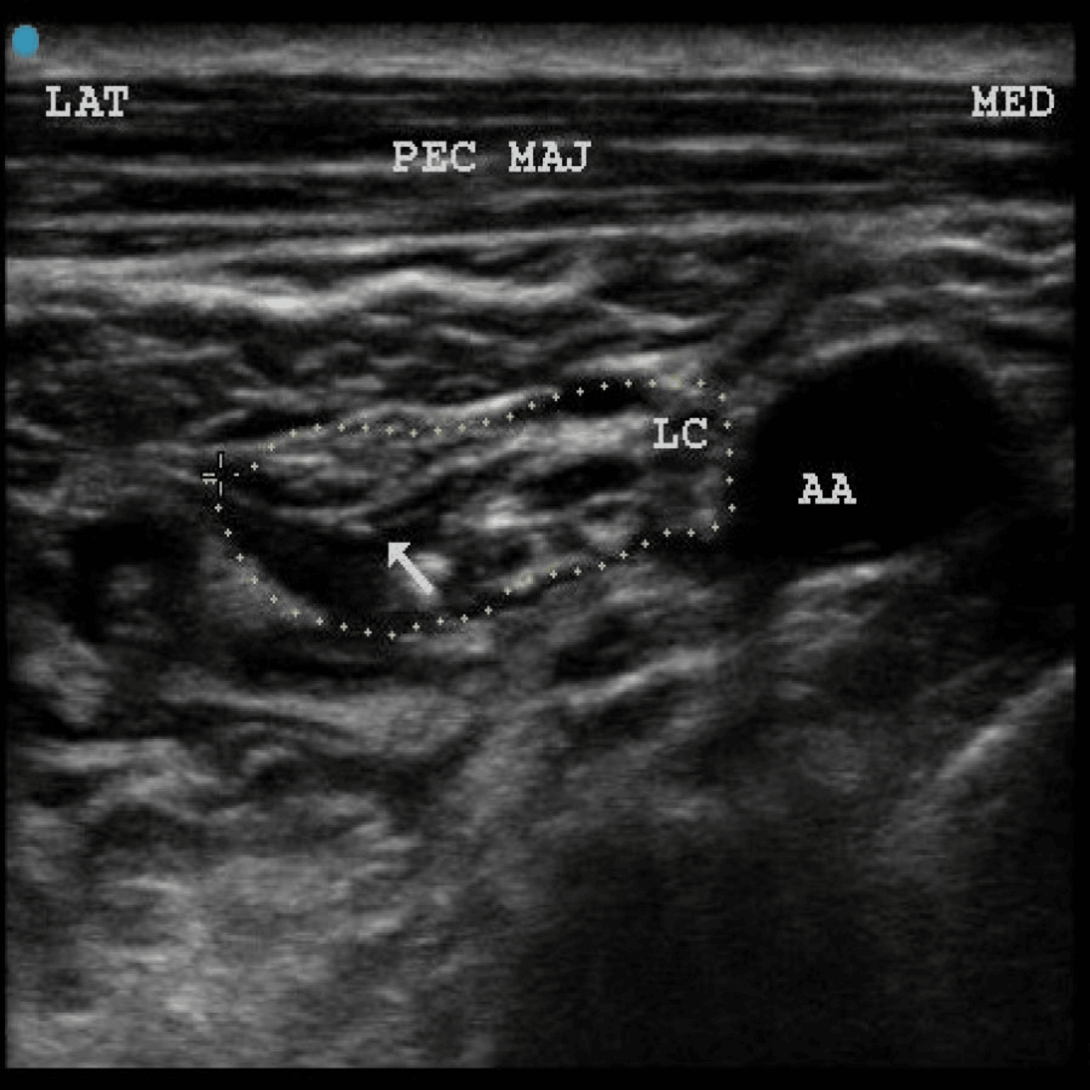
Dotted circle indicates brachial plexus cords with anechoic local anesthetic spread (indicated by white arrow). LAT: lateral; MED: medial; PEC MAJ: pectoralis major; AA: axillary artery; LC: lateral cord

Costoclavicular block: The ultrasound transducer was positioned inferior to the clavicle. The three cords were located lateral to the AA. After infiltrating the skin with 1 mL of 1% lidocaine, a 20-gauge, 5 cm block needle (Stimuplex Ultra 360; B. Braun Melsungen SE, Melsungen, Germany) was utilized for the CC-BPB. A block needle was attached to a peripheral nerve stimulator set at 0.5 mA, 0.1 milliseconds, and 1 Hz. Utilizing the previously mentioned procedure, the needle was introduced from the lateral to the medial direction in an in-plane manner, positioning the needle tip between the three cords (Figures 5-8) [7]. A medial (finger flexion) or posterior (elbow or wrist extension) cord motor response to peripheral nerve stimulation at <0.5 mA, with the needle tip positioned amid the nerve cluster, was deemed the preferred motor response.

Assessment of the Block

After LA administration through the block needle, measurements of the onset of sensory and motor blockade were taken by an independent observer who was blinded to the technique. Sensory blockade was graded according to a 3-point scale using a cold test with a spirit swab, as follows: 0, no block; 1, analgesia (the patient can feel touch but not cold); and 2, anesthesia (the patient cannot feel either touch or cold). Sensory blockade of the musculocutaneous, median, radial, and ulnar nerves was assessed on the lateral aspect of the forearm, the volar aspect of the thumb, the lateral aspect of the dorsum of the hand, and the volar aspect of the fifth finger, respectively. Motor blockade was graded on a 3-point scale: 0, no block; 1, paresis; and 2, paralysis. Motor blockade of the musculocutaneous, radial, median, and ulnar nerves was evaluated by elbow flexion, thumb abduction, thumb opposition, and thumb adduction, respectively. Overall, the maximal composite score was 16 points. The patient was considered ready for surgery when a minimal composite score of 14 points was achieved, provided the sensory block score was equal to or superior to 7 out of 8 points [7]. The onset of sensory or motor block is regarded as the time frame from the completion of the LA injection to the complete sensory or motor block. The sensory block duration was characterized as the timeframe from the administration of LA to the restoration of full sensation. Motor block duration was specified as the period from the administration of LA to the complete revival of motor strength. The duration of analgesia was defined as the period from the completion of LA to the first demand for analgesics.

The sensory and motor blocks were evaluated at three-minute intervals for 30 minutes following the administration of LA. Postoperative pain was assessed using a visual analogue scale (VAS) ranging from 0 to 10 points, with 0 indicating no pain and 10 representing the worst imaginable pain. Measurements were taken at one, two, four, six, 12, and 24 hours postoperatively. Patients exhibiting a VAS ≥ 4 were administered diclofenac sodium 75 mg IV as rescue analgesia. If the VAS score persisted at four or higher even after 30 minutes of diclofenac administration, an additional 50 mg IV diclofenac was administered. In cases of block failure in either approach, supplemental analgesia with intravenous (IV) administration of Inj. fentanyl in graded doses or conversion into general anesthesia was planned. Complications, such as nausea, vomiting, and local anesthetic systemic toxicity, vascular puncture, and nerve injury, were documented.

Outcome measures

The onset of motor block was the primary outcome. The secondary outcomes included onset of sensory block, block performance time, duration of sensory block, duration of motor block, time to the first request of rescue analgesia, duration of analgesia, VAS scores at one hour, two hours, four hours, six hours, 12 hours, and 24 hours postoperatively, duration of analgesia, patient satisfaction and incidence of side effects.

Statistical analysis

Data was analyzed using IBM SPSS Statistics for Windows, Version 27.0 (IBM Corp., Armonk, New York, United States). Continuous variables were tested for normality using the Shapiro-Wilk test and are presented as medians with interquartile ranges (IQR). Categorical variables are expressed as frequencies and percentages. Comparisons of demographic and baseline surgical characteristics between groups were performed using the Mann-Whitney U test for continuous variables and the chi-square test or Fisher’s exact test for categorical variables. Block characteristics (performance time, onset and duration of sensory and motor block, and duration of analgesia) were compared using the Mann-Whitney U test. Postoperative VAS scores were analyzed using the Friedman test for repeated measures within each group, followed by Bonferroni-adjusted pairwise comparisons to identify time points with significant differences. A p-value < 0.05 was considered statistically significant.

## Results

**Figure 9:**
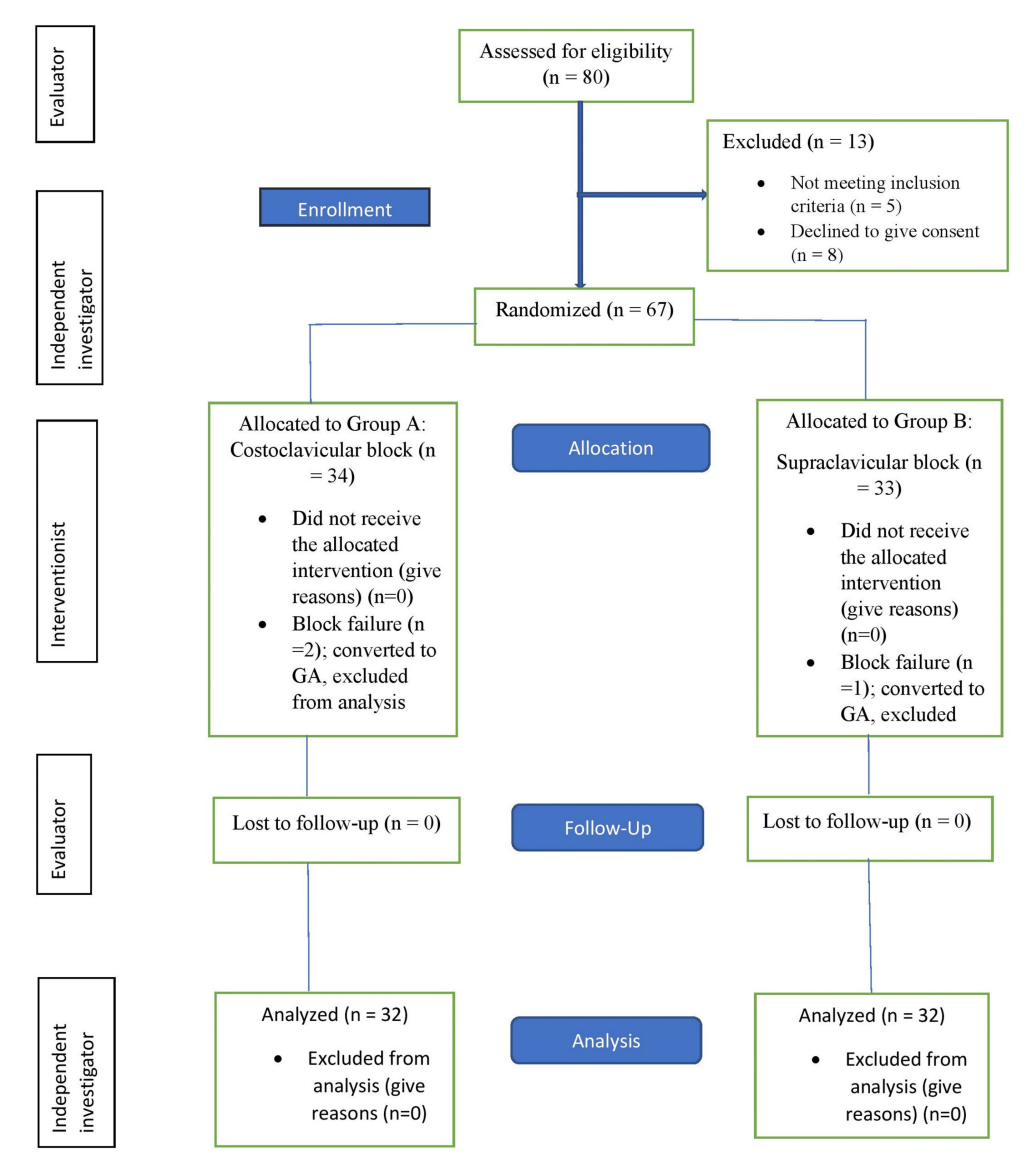
CONSORT flow diagram for enrolment, group allocation, follow-up, and analysis Eighty patients were assessed for eligibility. Of these, 13 were excluded (eight declined consent, five did not meet the inclusion criteria). The remaining 67 were randomized (34 in CC-BPB, 33 in SC-BPB). Three cases experienced block failure (two in CC-BPB, one in SC-BPB) and were excluded from the final analysis. Thus, 32 patients in each group completed the study. CONSORT: Consolidated Standards of Reporting Trials; GA: general anesthesia; CC-BPB: costoclavicular approach to brachial plexus block; SC-BPB: supraclavicular approach to brachial plexus block

A total of 80 subjects were assessed for eligibility, out of which eight declined to express consent, five couldn’t meet the inclusion criteria, and there was a block failure in three cases. A total of 32 patients from each group completed the study, with no loss to follow-up. Figure 9 illustrates the CONSORT (Consolidated Standards of Reporting Trials) diagram.

Both groups were comparable with respect to demographic variables, ASA physical status, and duration of surgery, with no statistically significant differences (Table 1).

**Table 1:** Demographic and surgical characteristics. The test statistic represents the value obtained from the Mann–Whitney U test for continuous variables and the chi-square test or Fisher’s exact test for categorical variables, as appropriate. ASA: American Society of Anesthesiologists

Parameters		Costoclavicular (n=32)	Supraclavicular (n=32)	Test Statistic	p-value
Age (years), median (IQR)		30 (23-42)	34 (25-46)	427.5	0.256
Sex, n (%)	Female	7 (21.9%)	11 (34.4%)	1.237	0.266
	Male	25 (78.1%)	21 (65.6%)		
Weight, median (IQR)		68 (60-77)	67 (59-74)	475.5	0.623
ASA, n (%)	1	20 (62.5%)	17 (53.1%)	0.577	0.448
	2	12 (37.5%)	15 (46.9%)		
Duration of surgery (minutes), median (IQR)		120 (99-135)	125 (102-133)	473.0	0.600

Block characteristics are summarized in Table 2. The block performance time was markedly shorter in the CC group (median difference −0.45 minutes, 95%CI −0.81 to 0.10, p < 0.001, r = 0.74), indicating a large effect size. Similarly, both the onset of sensory block (median difference −1.0 minutes, 95%CI −5.0 to 3.0, p = 0.0005, r = 0.42) and onset of motor block (median difference −1.0 minutes, 95%CI −5.0 to 2.0, p = 0.0011, r = 0.40) occurred significantly faster in the CC group, reflecting large and clinically relevant effects. In contrast, there were no significant differences in the duration of sensory block (median difference −4.0 minutes, 95%CI −279.7 to 264.3, p = 0.687, r = 0.05), duration of motor block (median difference −4.0 minutes, 95%CI −354.3 to 350.0, p = 0.559, r = 0.07), or duration of analgesia (median difference −5.0 minutes, 95%CI −266.0 to 250.0, p = 0.550, r = 0.08) between the two groups. The narrow effect sizes and confidence intervals encompassing zero indicate negligible clinical differences. These findings suggest that while the costoclavicular approach offers faster block onset and shorter procedural time, both techniques provide comparable block duration and postoperative analgesic efficacy.

**Table 2:** Block characteristics between the two groups Data are presented as median (interquartile range, IQR). Median differences are expressed as Hodges–Lehmann estimates with 95% confidence intervals (CIs). Exact p-values and effect sizes (r) were derived using the Mann–Whitney U test (r = Z / √N, N = 64). According to Cohen’s conventions, r = 0.1 (small), 0.3 (medium), and 0.5 (large).

Variable	Costoclavicular (n=32)	Supraclavicular (n=32)	Median Difference (Hodges–Lehmann)	95% CI	Test Statistic	p-value	Effect size (r)
Block performance time (min)	1.53 (1.37-1.71)	1.98 (1.90-2.10)	−0.45	−0.81 to 0.10	72.0	0.000	0.74
Onset of sensory block (min)	9 (9-10)	10 (10-11)	−1.00	−5.0 to 3.0	263.5	0.001	0.42
Onset of motor block (min)	12 (12-15)	13 (13-16)	−1.00	−5.0 to 2.0	275.5	0.001	0.40
Duration of sensory block (min)	840 (805-900)	847 (809-901)	−4.00	−279.7 to 264.3	481.5	0.682	0.05
Duration of motor block (min)	765 (720-790)	767 (723-799)	−4.00	−354.3 to 350.0	468.0	0.554	0.07
Time until request of first analgesia (min)	1440 (1440-1440)	1440 (1440-1440)			464.0	0.285	
Duration of analgesia (min)	915 (850-960)	941 (853-977)	-5.00	−266.0 to 250.0	467.0	0.545	0.07

**Figure 10:**
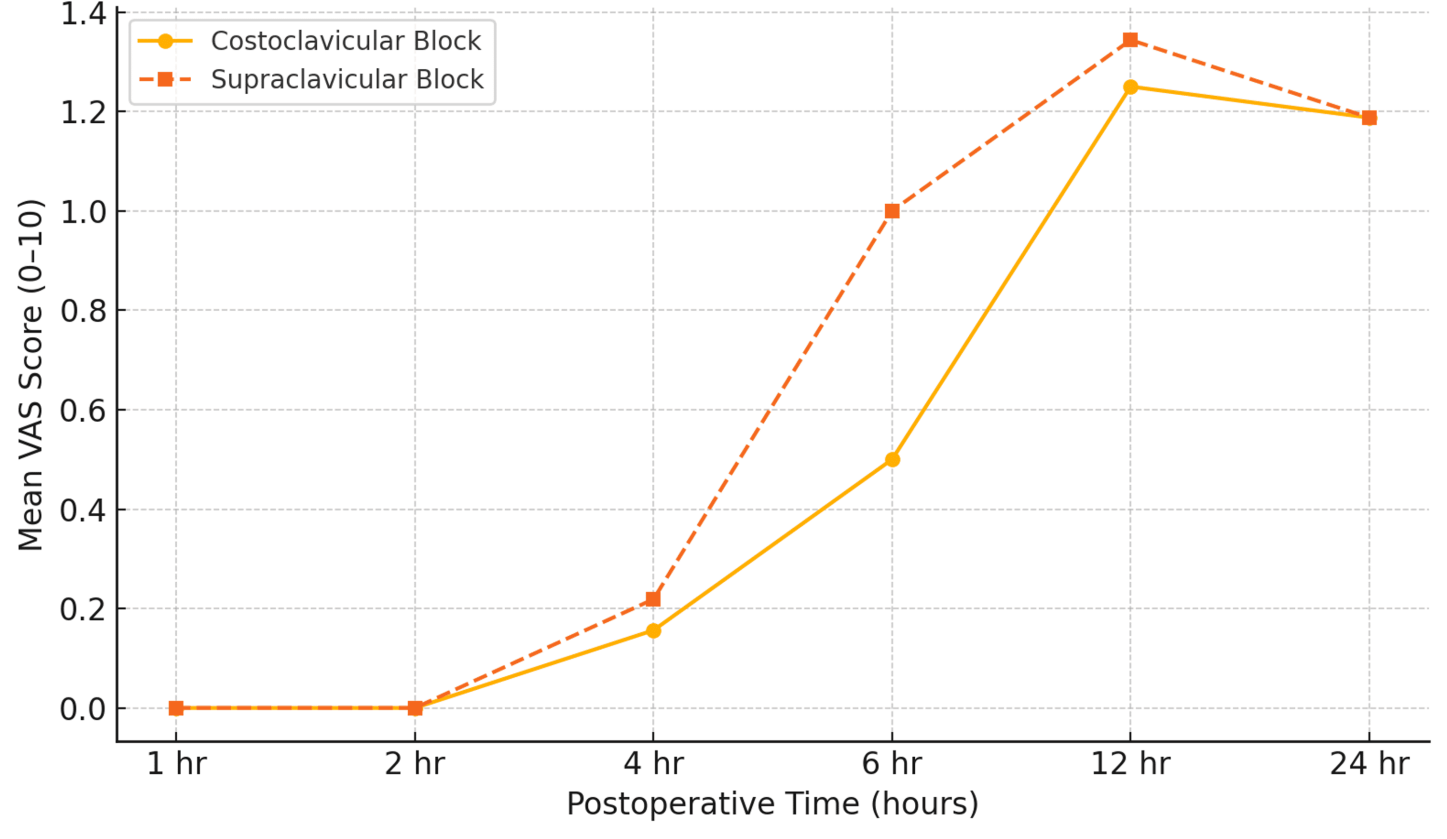
Postoperative pain trends (VAS scores) over 24 hours. Mean postoperative visual analogue scale (VAS) scores (0–10) recorded at one, two, four, six, 12, and 24 hours following surgery for the costoclavicular (solid line, circles) and supraclavicular (dashed line, squares) brachial plexus block groups. Both groups demonstrated near-zero pain scores up to 4 hours postoperatively, with gradual increases noted at 6–12 hours and mild pain levels at 24 hours.

Both groups maintained near-zero pain scores for the first four hours postoperatively, with mild increases at 6-12 hours and stabilized by 24 hours. The trend lines nearly overlap, confirming comparable postoperative analgesic profiles between costoclavicular and supraclavicular approaches (Figure 10).

Postoperative pain scores, as assessed by the VAS scale, are shown in Appendix A. Both groups demonstrated excellent analgesia in the early postoperative period (one to four hours), with no patient reporting pain. At six hours, differences emerged, with a higher proportion of CC patients reporting lower VAS scores compared to the SC group. At 12 and 24 hours, both groups exhibited mild pain levels, with no clinically significant differences between them. The Friedman test demonstrated a substantial change in VAS scores over time within both groups (p < 0.001). Pairwise comparisons in Appendix B revealed significant increases in VAS scores between six and 12 hours and between 12 and 24 hours, consistent with the gradual wearing off of the block effect.

## Discussion

This randomized controlled trial compared the clinical efficacy, block dynamics, and postoperative outcomes of ultrasound-guided CC-BPB and SC-BPB in patients undergoing forearm and hand surgeries. Our findings demonstrate that CC-BPB offers significantly shorter block performance times and a faster onset of both sensory and motor blockade compared to the SC approach. However, the duration of sensory and motor blocks, postoperative analgesia, pain scores, and incidence of complications were comparable between the two groups. While these differences are statistically significant, their clinical relevance lies in the potential operational efficiency they confer. Faster block onset can facilitate smoother operating room turnover, reduce anesthesia preparation time, and improve workflow in busy or time-sensitive surgical settings, without compromising safety or analgesic duration.

Although the absolute difference in onset times between the two approaches was approximately one minute, this finding should be interpreted in the context of procedural efficiency. In regional anesthesia practice, even small but consistent reductions in onset and performance time can translate into improved operating room workflow, reduced anesthesia preparation time, and greater patient throughput in high-volume settings. The large effect sizes observed in our analysis further suggest that the difference, while numerically modest, reflects a consistent physiological advantage of the CC approach rather than a chance finding. Although the absolute reduction in combined block performance and onset times was modest (approximately 90 seconds), this difference consistently favored the CC approach across all measured block characteristics. While unlikely to influence clinical outcomes in routine cases, such reproducible efficiency may be advantageous in settings where time-sensitive anesthesia is critical, such as high-volume surgical lists, day-care procedures, and trauma cases requiring rapid turnover.

Numerous studies have been conducted to compare the SC approach with the IC approach for BPBs [9]. Additionally, trials were conducted to compare the CC approach with the paracoracoid and lateral sagittal approaches for IC blocks [10]. Our study is unique in the sense that we have compared the SC technique with the newer CC technique. We have combined the nerve stimulation technique along with ultrasound guidance to improve the efficacy of the blocks.

The reduced block performance time observed with CC-BPB is likely attributable to the clustered anatomical arrangement of the three cords of the brachial plexus lateral to the axillary artery in the CC space. This predictable and compact neural configuration facilitates a single-injection technique and rapid spread of the local anesthetic, thereby shortening the procedural time. In contrast, SC-BPB typically requires multiple injections around the SC artery to achieve complete blockade, resulting in a relatively longer performance time. Our results are consistent with prior studies that have reported faster block execution using the CC approach [8,11,12]. However, some studies, such as those by Zhang et al. [13] and Luo et al. [14], found no significant advantage, likely due to differences in technique (e.g., modified double-injection) or retrospective study design.

The volume of LA used in our trial was 20 ml of 0.5% ropivacaine based on previous recommendations [15]. The significantly faster onset of sensory and motor blocks with CC-BPB in our study further supports the anatomical advantages of this approach. The cords of the brachial plexus in the CC space are consistently aligned and in proximity, allowing efficient local anesthetic diffusion. Ramesh et al. [8] and Devi et al. [11] reported similar observations. Li et al. similarly noted that the onset of sensory and motor blocks occurred within an average of five minutes, with a range of 5-15 minutes for sensory blockade and 5-10 minutes for motor blockade [7]. This rapid onset can be particularly advantageous in clinical settings where expedited surgical readiness is essential. Although the differences in sensory and motor onset times between the CC-BPB and SC-BPB groups were statistically significant (approximately one minute faster with CC-BPB), the clinical significance of this difference should be interpreted with caution. For routine elective surgeries, a one-minute reduction may not substantially alter patient outcomes. Nevertheless, in high-volume or resource-limited settings, where operating room efficiency and turnover times are critical, even marginal reductions in block onset and performance time can enhance workflow efficiency and anesthesia readiness

Despite these differences in onset and performance times, both approaches provided similar durations of sensory and motor blockade, as well as postoperative analgesia. These findings align with multiple studies [8,11,14] and suggest that the spread and pharmacodynamics of the local anesthetic are ultimately comparable once the block is established. Notably, Zhang et al. reported longer block duration and analgesia with CC-BPB, but the retrospective nature of their study may limit the strength of their conclusions [13]. Our VAS pain scores and time to first analgesic request were also similar across groups, indicating equivalent clinical efficacy in postoperative pain control.

Importantly, no significant adverse events were reported in either group, reinforcing the safety profile of both techniques. The costoclavicular approach may offer a theoretical advantage in reducing risks such as pleural puncture and pneumothorax, as the needle trajectory is directed away from the pleura and major vessels. Additionally, CC-BPB may minimize the risk of phrenic nerve involvement and subsequent hemidiaphragmatic paralysis, a complication more commonly associated with the SC approach [3,4]. However, our study did not directly evaluate diaphragmatic function, which remains an important area for future investigation.

Limitations

There are certain limitations to our trial. The relatively small sample size and single-center design may limit the generalizability of our findings. Furthermore, we did not assess the incidence of phrenic nerve blockade or diaphragmatic function, which could offer additional information about safety. Future multicenter trials with larger sample sizes and objective respiratory assessments are warranted to validate these findings and further delineate the safety profile of CC-BPB. The incidence of hemidiaphragmatic paralysis was not assessed.

## Conclusions

Ultrasound-guided CC-BPB offers significant procedural advantages over the traditional supraclavicular approach for forearm and hand surgeries. It provides a faster onset of both sensory and motor blockade and a shorter block performance time, with large effect sizes indicating clinically meaningful efficiency gains. Importantly, these benefits are achieved without compromising block duration, postoperative analgesia, or safety.

Clinically, the CC approach may be preferred in time-sensitive or high-turnover surgical settings, where faster anesthesia onset can enhance operating room efficiency and patient throughput. Given its favorable safety profile and comparable analgesic duration, the CC-BPB represents a reliable and practical alternative to the SC technique in routine anesthesia practice. Future multicenter trials incorporating diaphragmatic function and long-term outcomes are warranted to confirm these findings and further establish its clinical superiority.

## Appendices

Appendix A 

**Table 3:** Postoperative VAS score between the two groups. Related-samples Friedman's two-way analysis of variance (ANOVA) by Ranks Summary. The test statistic represents the Friedman test statistic for repeated measures within each group. VAS: visual analog scale

VAS		Group			
		Costoclavicular (n=32)		Supraclavicular (n=32)	
		Count (%)	Mean Rank	Count (%)	Mean Rank
1 hour	0	32 (100%)	2.22	32 (100%)	1.92
2 hours	0	32 (100%)	2.22	32 (100%)	1.92
4 hours	0	27 (84%)	2.64	25 (78%)	2.50
	1	5 (16%)		7 (22%)	
6 hours	0	17 (53%)	3.55	2 (6%)	4.66
	1	14 (44%)		29 (91%)	
	2	1 (3%)		0 (0%)	
	3	0 (0%)		1 (3%)	
12 hours	0	2 (6%)	5.13	0 (0%)	5.06
	1	26 (81%)		27 (84%)	
	2	1 (3%)		2 (6%)	
	4	3 (9%)		3 (9%)	
24 hours	1	28 (88%)	5.25	28 (88%)	4.94
	2	2 (6%)		2 (6%)	
	3	2 (6%)		2 (6%)	
Test Statistic		124.662		139.671	
p-value		<0.001		<0.001	

Appendix B 

**Table 4:** Pairwise comparisons of VAS score at each time pair. The test statistic represents the standardized test statistic from pairwise post hoc comparisons following the Friedman test. P-values are Bonferroni-adjusted for multiple comparisons. Each row tests the null hypothesis that the distributions of Sample 1 and Sample 2 are the same. Asymptotic significances (2-sided tests) are displayed. The significance level is .050. ^a^ Significance values have been adjusted by the Bonferroni correction for multiple tests. HR: hours; VAS: visual analog scale

Group	Pairwise Comparisons	Test Statistic	Std. Error	Std. Test Statistic	p-value	Adj. p-value^a^
Costoclavicular	VAS 1 HR-VAS 2 HR	0.000	0.468	0.000	1.000	1.000
	VAS 1 HR-VAS 4 HR	-0.422	0.468	-0.902	0.367	1.000
	VAS 1 HR-VAS 6 HR	-1.328	0.468	-2.840	0.005	0.068
	VAS 1 HR-VAS 12 HR	-2.906	0.468	-6.214	0.000	0.000
	VAS 1 HR-VAS 24 HR	-3.031	0.468	-6.481	0.000	0.000
	VAS 2 HR-VAS 4 HR	-0.422	0.468	-0.902	0.367	1.000
	VAS 2 HR-VAS 6 HR	-1.328	0.468	-2.840	0.005	0.068
	VAS 2 HR-VAS 12 HR	-2.906	0.468	-6.214	0.000	0.000
	VAS 2 HR-VAS 24 HR	-3.031	0.468	-6.481	0.000	0.000
	VAS 4 HR-VAS 6 HR	-0.906	0.468	-1.938	0.053	0.790
	VAS 4 HR-VAS 12 HR	-2.484	0.468	-5.312	0.000	0.000
	VAS 4 HR-VAS 24 HR	-2.609	0.468	-5.579	0.000	0.000
	VAS 6 HR-VAS 12 HR	-1.578	0.468	-3.374	0.001	0.011
	VAS 6 HR-VAS 24 HR	-1.703	0.468	-3.641	0.000	0.004
	VAS 12 HR-VAS 24 HR	-0.125	0.468	-0.267	0.789	1.000
Supraclavicular	VAS 1 HR-VAS 2 HR	0.000	0.468	0.000	1.000	1.000
	VAS 1 HR-VAS 4 HR	-0.578	0.468	-1.236	0.216	1.000
	VAS 1 HR-VAS 6 HR	-2.734	0.468	-5.846	0.000	0.000
	VAS 1 HR-VAS 12 HR	-3.141	0.468	-6.715	0.000	0.000
	VAS 1 HR-VAS 24 HR	-3.016	0.468	-6.448	0.000	0.000
	VAS 2 HR-VAS 4 HR	-0.578	0.468	-1.236	0.216	1.000
	VAS 2 HR-VAS 6 HR	-2.734	0.468	-5.846	0.000	0.000
	VAS 2 HR-VAS 12 HR	-3.141	0.468	-6.715	0.000	0.000
	VAS 2 HR-VAS 24 HR	-3.016	0.468	-6.448	0.000	0.000
	VAS 4 HR-VAS 6 HR	-2.156	0.468	-4.610	0.000	0.000
	VAS 4 HR-VAS 12 HR	-2.563	0.468	-5.479	0.000	0.000
	VAS 4 HR-VAS 24 HR	-2.438	0.468	-5.212	0.000	0.000
	VAS 6 HR-VAS 12 HR	-0.406	0.468	-0.869	0.385	1.000
	VAS 6 HR-VAS 24 HR	-0.281	0.468	-0.601	0.548	1.000
	VAS 24 HR-VAS 12 HR	0.125	0.468	0.267	0.789	1.000
